# Assistive Technologies for Individuals with a Disability from a Neurological Condition: A Narrative Review on the Multimodal Integration

**DOI:** 10.3390/healthcare13131580

**Published:** 2025-07-01

**Authors:** Mirjam Bonanno, Beatrice Saracino, Irene Ciancarelli, Giuseppe Panza, Alfredo Manuli, Giovanni Morone, Rocco Salvatore Calabrò

**Affiliations:** 1 IRCCS Centro Neurolesi Bonino-Pulejo, Via Palermo, Cda Casazza, SS 113, 98124 Messina, Italy; mirjam.bonanno@irccsme.it (M.B.); roccos.calabro@irccsme.it (R.S.C.); 2 Department of Life, Health and Environmental Sciences, University of L’Aquila, 67100 L’Aquila, Italy; beatrice.saracino@student.univaq.it (B.S.); irene.ciancarelli@univaq.it (I.C.); 3 Dipartimento delle Scienze Mediche e della Lungodegenza, U.O.C. di Medicina Interna A.O.R.N. “San Pio”, P.O. “G. Rummo”, 82100 Benevento, Italy; giuseppepanza80@gmail.com; 4 Physical Medicine and Rehabilitation Unit, AOU Policlinico Universitario in Messina, 98125 Messina, Italy; manulialfredo@gmail.com; 5 Clinical Laboratory of Experimental Neurorehabilitation, Santa Lucia Foundation-IRCCS, 00179 Rome, Italy

**Keywords:** assistive devices, assistive technology, robot, brain–computer interfaces, neuroprosthesis, health technology assessment

## Abstract

Background/Objectives: Neurological disorders often result in a broad spectrum of disabilities that impact mobility, communication, cognition, and sensory processing, leading to significant limitations in independence and quality of life. Assistive technologies (ATs) offer tools to compensate for these impairments, support daily living, and improve quality of life. The World Health Organization encourages the adoption and diffusion of effective assistive technology (AT). This narrative review aims to explore the integration, benefits, and challenges of assistive technologies in individuals with neurological disabilities, focusing on their role across mobility, communication, cognitive, and sensory domains. Methods: A narrative approach was adopted by reviewing relevant studies published between 2014 and 2024. Literature was sourced from PubMed and Scopus using specific keyword combinations related to assistive technology and neurological disorders. Results: Findings highlight the potential of ATs, ranging from traditional aids to intelligent systems like brain–computer interfaces and AI-driven devices, to enhance autonomy, communication, and quality of life. However, significant barriers remain, including usability issues, training requirements, accessibility disparities, limited user involvement in design, and a low diffusion of a health technology assessment approach. Conclusions: Future directions emphasize the need for multidimensional, user-centered solutions that integrate personalization through machine learning and artificial intelligence to ensure long-term adoption and efficacy. For instance, combining brain–computer interfaces (BCIs) with virtual reality (VR) using machine learning algorithms could help monitor cognitive load in real time. Similarly, ATs driven by artificial intelligence technology could be useful to dynamically respond to users’ physiological and behavioral data to optimize support in daily tasks.

## 1. Introduction

Neurological disorders (NDs) can derive from the combination of biological, genetic, and environmental factors (e.g., congenital abnormalities, infections, and traumatic injuries) that affect the central nervous system. NDs are a common cause of impaired mobility (i.e., muscle weakness, paralyses, spasticity), speech alterations (i.e., from aphasia to lack of communication), sensation (i.e., paresthesia, hypoesthesia, allodynia), and cognitive functioning (i.e., executive functions, memory, attention processes, etc.) [1,2]. NDs include a wide range of conditions such as acquired brain injury including stroke and traumatic brain injury (TBI), spinal cord injury (SCI), neurodegenerative disorders, including multiple sclerosis (MS), Parkinson’s disease (PD), Alzheimer’s disease (AD), and lateral amyotrophic sclerosis (ALS) [3]. All these pathologies share the presence of multiple disabilities, varying in severity from mild to severe. These impair functional independence, affecting daily activities, social participation, quality of life, and caregivers’ burden [4,5]. After an acute brain injury, the patient needs to be stabilized. However, symptoms derived from central nervous system damage often persist, leading to reduced functional independence and a decline in quality of life [6]. On the other hand, patients affected by neurodegenerative disorders tend to progressively lose functional independence, as the disease leads to a progressive decline, thus needing continuous assistance [7].

In this context, assistive technologies (ATs) can play a critical role in improving the quality of life of both disabled individuals and their caregivers, promoting rehabilitation efficacy, promoting independence, and enabling individuals to participate more in daily activities [8,9]. The integration of AT for individuals with chronic disabilities has made significant advances in various medical fields, and the rehabilitation of subjects with NDs is no exception [8]. In general, AT is defined as “any item, piece of equipment, or product system, whether acquired commercially off the shelf, modified, or customized, that is used to increase, maintain, or improve functional capabilities of individuals with disabilities” [10]. AT encompasses devices, equipment, and tools designed to promote independence and self-determination for neurological patients [11]. According to learning principles, specifically, the causal link between behavioral responses and environmental consequences, an AT-based program serves as a functional bridge between an individual’s residual abilities and the high demands of their environment [11]. Different from devices used for rehabilitative purposes, ATs aim to aid individuals performing daily life activities by compensating for lost or residual functions [12].

In recent years, research in the field of AT has made great developments in terms of technological innovations, such as brain–computer interfaces, neuroprosthetics as well as ocular communicators [13]. Nowadays, ATs can assist in a variety of patient functions, from mobility to speech, and senses. Among assistive technologies, an even more specific area has benefited from great attention and has undergone considerable expansion, and is that of digital assistive technology [14]. For example, ATs, using virtual reality, can be used as a valuable strategy in mitigating challenges with remote communication [11]. This type of intervention could improve social interactions and mental well-being, avoiding isolation in patients with NDs and communication difficulties. Despite their innovative nature, AT devices still face several challenges, including usability, customer satisfaction, cost reduction, and integration into home, work, and social environments [15].

Other reviews on ATs in the ND population have addressed a variety of aspects. For example, Copolillo and Ivanoff [16] highlighted the importance of ATs for individuals with neurovisual impairments, suggesting the use of prisms and telescopic lenses to enhance visual attention and reduce the impact of visual field deficits. However, the authors emphasized a substantial need for further research on the specific needs of individuals with vision impairments resulting from neurological conditions. In another review, Alam et al. [17] identified the most commonly used assistive devices in the neurological population. They suggested that such devices can be effectively employed by physiotherapists to optimize rehabilitation outcomes. Some assistive devices support both therapeutic interventions and daily living activities, contributing to the maintenance or improvement of quality of life. However, appropriate use is critical, as overuse or misuse may lead to increased patient dependency and negatively impact the rehabilitation process. More randomized controlled trials (RCTs) are needed to better define the role and efficacy of assistive devices in neurological rehabilitation. A recent position paper by Stasolla et al. [11] proposes that the integration of innovative technologies, such as virtual reality and remote systems, within AT frameworks represents a promising approach to support cognitive impairments in individuals with neurological disorders. This combined technology-assisted strategy could promote active engagement, immersive experiences, and meaningful participation in simulated real-life contexts, while also encouraging the involvement of caregivers and families. While previous reviews in this field have addressed isolated aspects of ATs, the present review offers a comprehensive perspective by examining their application across multiple functional domains in individuals with NDs.

In this context, our review explores the current state of integration of ATs in subjects with neurological disabilities, highlighting advances, challenges, and potential for future developments. To this aim, the paper is divided into several sections: Section 2 describes materials and methods. Section 3 reports a detailed overview of different categories of ATs used in neurological disorders, organized according to functional domains: mobility aids, communication tools, cognitive support systems, sensory aids, brain–computer interfaces, and home and environmental control technologies. Furthermore, the paper discusses, in Section 4, current challenges and limitations in AT applications, including usability, accessibility, and patient acceptance, and presents potential solutions to address these issues.

## 2. Materials and Methods

Given the narrative nature of the paper, we have only reported the most relevant papers in this field of research by searching PubMed and Scopus. We considered the last 10 years (2014–2024), and searched for title/abstract, using a combination of the following keywords for both databases: (“assistive technologies” OR “assistive devices” OR “rehabilitation technology” OR “assistive tools”) AND (“neurology” OR “neurological disorders” OR “neurorehabilitation” OR “brain injury” OR “stroke” OR “Parkinson’s disease” OR “multiple sclerosis”) AND (“disability” OR “disabled persons” OR “disabled people” OR “people with disabilities”) AND (“integration” OR “implementation” OR “adoption” OR “use” OR “impact”).

This narrative review was conducted to explore the role and integration of ATs in individuals with neurological disabilities. The review was guided by the following research question:

**RQ1.**  What is the current state of integration, application, and limitations of ATs in individuals with neurological disabilities, and what future directions can be identified for their effective implementation across functional domains?

To answer this question, we selected studies based on their relevance to the topic and quality of evidence. The inclusion criteria were: (i) studies published between 2014 and 2024; (ii) articles written in English; (iii) pilot studies, observational studies, randomized controlled trials, case–control studies, and systematic reviews; and (iv) studies focused on ATs in NDs. References cited within selected articles were also considered. Exclusion criteria included: (i) studies not related to assistive technologies or neurological populations; (ii) non-peer-reviewed articles; and (iii) conference abstracts, editorials, commentaries, and articles lacking sufficient methodological detail. Each article was evaluated through its title, abstract, text, and scientific validity [18].

## 3. Applications and Integration of Assistive Technologies in Neurological Disorders

In this section, we reported a detailed overview of different categories of ATs used in NDs, organized according to functional domains: mobility, communication, cognitive, sensory aids, brain–computer interfaces, home and environmental control technologies.

### 3.1. Mobility Aids

Mobility aids, such as canes, crutches, walkers, and wheelchairs, are essential in improving the autonomy of people with motor impairments. The adoption of these aids helps reduce dependence on human assistance and promotes social participation, enhancing the users’ quality of life. However, the effectiveness of these aids depends on various factors, including accessibility, aid customization, and the support received for their use.

The importance of assistive aids’ accessibility was analyzed in a study conducted in Chile by Hirmas-Adauy et al. (2016) [19], which evaluated the effectiveness of a national policy to distribute assistive devices to elderly people with limited mobility. The longitudinal study followed 309 patients for 7 months, analyzing the devices’ impact on their independence and quality of life. The results obtained showed a significant improvement in mobility and autonomy in daily activities, along with a reduction in the risk of falls and pain. Furthermore, the level of satisfaction among beneficiaries was extremely high (100% satisfaction). Nonetheless, some issues emerged: only 2% of patients received adequate follow-up, and the assistance program covered only 25% of the estimated need, highlighting the need for greater accessibility and continuous monitoring of the effectiveness of the aids over time [19].

Moreover, the role of walking aids was also examined in a systematic review conducted by Bertrand et al. (2017) [20], which explored their role in improving participation and activity for adults with physical disabilities. The study highlighted that these tools promote mobility and quality of life, but they may present limitations related to their physical characteristics, environmental adaptation challenges, and personal reluctance to adoption. Despite the use of traditional, there is a growing interest in the adoption of innovative AT. For instance, exoskeletons and robotic devices are designed to restore walking ability in individuals with SCI or other NDs. Rather than relying on rehabilitation training to gradually restore lost motor function, assistive exoskeletons are designed solely to provide support, enabling individuals to maintain independence in daily activities regardless of their impairments. A clinical study conducted in the United States by Evans et al. (2015) [21] evaluated the effectiveness of the Indego exoskeleton on patients with spinal injuries. After five training sessions, participants improved their walking speed and distance, demonstrating the potential of these devices in supporting walking rehabilitation. Nevertheless, the spread of exoskeletons is still limited by high costs and the need for specific training for users and healthcare providers [21]. In this sense, Morris et al. [22] have highlighted that a wide range of patients and physiotherapists expressed concerns about rigid and soft exoskeletons (for a review, see Siviy et al. [23]). Specifically, rigid exoskeletons (e.g., Indego) may force patients into unnatural movement patterns or even erroneously detect and force movements, for instance, interpreting the patient as trying to sit when they are walking [24,25]. Similar constraints may also be present in other lower-limb orthoses, such as ankle–foot orthoses and knee–ankle–foot orthoses. However, these devices are generally intended for different purposes, such as supporting dorsiflexion while allowing relative freedom in plantar flexion. In the case of rigid exoskeletons, such constraints may be more evident due to the mechanical guidance provided by the device, particularly when used by individuals with severe motor impairments who are unable to walk independently [26].

From an engineering point of view, the first challenge is to develop a lightweight exoskeleton with soft actuators equipped with a user-friendly interface. Additionally, they need to be designed to fit within a wearable, garment-like suit while ensuring comfort. The stiffness level directly influences the force transmitted to the human body, making it essential to balance rigidity with assistive effectiveness. For instance, compliant actuators offer a combination of safety, comfort, and portability due to their lightweight design. However, they may have lower bandwidth or peak force compared to traditional, heavier rigid actuation technologies like motors and gears [27]. These considerations have important implications in the clinical context when advising the patient of the specific AT, especially to facilitate the function of the lower limbs. In this context, the Ekso (Ekso Bionics, CA, USA), a rigid exoskeleton, requires at least a week of therapist training and must initially be used under supervision [25,28].

Future devices should be more efficient, minimizing both training requirements and the time needed for effective use. Differently is for the upper limb, in which the assistive hand exoskeletons are designed to support grasping and manipulating functions. These devices provide external mechanical support to help people perform activities of daily life [29]. According to Hays et al. [29], the potential challenges for the design of upper-limb ATs are the complex anatomical and biomechanical structure of the shoulder and hand. To assist joint movement during abduction and adduction, exoskeletons must precisely mimic natural joint motions to prevent misalignment issues and enhance kinematic compatibility. Some studies [30] suggest using spherical mechanisms to replicate the three degrees-of-freedom in shoulder and wrist movements, assuming they function as ball-and-socket joints. However, this method may affect the instantaneous center of rotation within these joints. Moreover, the thumb remains the most complex structure, with its kinematic movement modeling still not fully defined (Hayes). Moreover, Gandolla et al. [31] have proposed and tested an AT device to improve the quality of life for people with severe upper-limb impairments. In this context, passive and semi-active solutions are not effective with severely impaired people who have no residual muscular force in their arms. In addition, force-based and EMG-based devices are not suitable for patients with very low residual force. To fill this gap, these authors developed the BRIDGE exoskeleton, which is a four-degree-of-freedom upper limb fully active exoskeleton that can be driven by a very sensitive joystick or by a vocal interface. This device was tested on patients with muscular dystrophy, in which increased functional activity was noted as well as overall “excellent” usability. The use of assistive mobility devices has proven effective in improving the independence and quality of life of people with motor impairments. The studies considered confirming that canes and crutches can reduce the need for human assistance, while devices like walkers and wheelchairs complement this support. However, significant challenges regarding coverage of needs, monitoring the effectiveness of aids, and providing adequate support to users remain.

### 3.2. Communication Aids

Disabilities as well as conditions such as stroke, ALS, and disorders of consciousness like locked-in syndrome can severely impair the ability to speak and communicate. In such cases, augmentative and alternative communication (AAC) devices play a crucial role in restoring communication abilities [32]. AAC comprises tools and strategies that aim to support or replace verbal communication and to improve the quality of life for individuals with communication disabilities [33]. These devices can be categorized into three types based on the technology used: (i) low-tech AAC, including non-electronic tools (e.g., gesture vocabulary, memory book, communication passport, visual schedule and visual strips, environmental labeling, communication boards, modified books); (ii) mid-tech AAC, such as electronic tools that do not require a computer (voice output communication aids, symbolic and alphabetic communicators); and (iii) high-tech AAC, like dynamic communicator, communication software, eye-tracking devices.

Eye-tracking devices are useful for people affected by ALS, since the disease determines a constant progressive loss of voluntary strength. In advanced phases of the disease, motor and phonatory impairments can limit patients’ communication abilities. Caligari et al. [34] investigated the effects of using a high-tech eye-tacker device and eye-transfer board as an AAC in people with ALS. They found that the eye-transfer board significantly improved the communicative abilities compared to the condition without the device, but a further improvement was obtained with the eye-tracker device. Additionally, this promoted higher levels of user satisfaction, with an increase in the quality of life. It is worth noting that high-tech AAC devices in people with ALS should be used to maintain caregiver-independent communication and environmental control even in the advanced disease phases. Thus, they enable patients to preserve social participation. In this context, an aspect that should not be neglected is that AAC timing based on the natural course of neurodegenerative disease is pivotal. In fact, the training should start early to ensure adequate time for AAC education and device acquisition. Patients and their families will consider AAC support as an effective communication tool only if they learn to incorporate it into everyday conversations [35]. Communication supports, whether high-tech, low-tech, or no-tech, should be customized to each individual’s needs and abilities and adjusted as the disease progresses [2].

### 3.3. Cognitive Support Tools

In the field of cognitive support tools, ATs include memory aids, reminder systems, and specialized software, and can be valuable support for individuals who have to face these challenges. In the last few years, the utilization of technology in the assistance of people with cognitive difficulties has marked a major advancement in neuropsychological rehabilitation [36]. Cognitive support tools allow individuals with cognitive processing issues to benefit from greater autonomy, helping facilitate daily activities in real-life environments. Moreover, such tools can reduce stress and the workload of caregivers, promoting greater independence [37]. An additional significant advantage is the potential to bridge the “digital divide,” making technology more accessible to patients with cognitive disabilities [36]. In this sense, a study [38] examines how the world’s population is aging rapidly, with a significant increase in functional limitations among the elderly, inevitably causing a higher caregiving burden.

ATs, which enhance the independence and quality of life of the elderly, have proven effective in reducing the load on caregivers. These technologies help save caregivers’ time and resources, decreasing the energy and level of assistance required, reducing anxiety and the risk of injury, and improving the user’s safety and independence. Despite the benefits, some caregivers express concerns about potential limitations of assistive technologies, fearing they may add further stress or complications [39].

Nonetheless, most studies suggest that these technologies help reduce the overall burden by facilitating daily activities. The reported study [40] has shown that external aid is effective in improving the independence and participation of individuals with cognitive deficits in daily life. A 20-year-long study of technological tools for cognition highlighted that these solutions enable individuals with acquired brain injuries (ABIs) to perform activities that would otherwise be difficult or impossible [41].

In particular, cognitive support tools range from simple solutions to more advanced technological devices: (i) low-tech tools, which include practical and easy-to-use aids, such as writing down appointments on paper or using a tray to organize daily medications; and (ii) high-tech tools, which offer advanced and often automated features like smartphone apps that send bill payment reminders, electronic calendars for scheduling appointments, or virtual assistants that help manage daily tasks. The main difference between low-tech and high-tech tools lies in their automated functions and the complexity of tasks that can be performed. Both types support many aspects of daily life, ranging from personal care to household management, and also include school and work activities, contributing to the improvement of autonomy and efficiency in everyday tasks [40].

Further research is needed to better understand the challenges associated with the use of these technologies and to assess how they can be improved and made even more useful and effective. Furthermore, it is pivotal to pay more attention to supporting informal caregivers, as their role is crucial in enhancing the quality of life of the elderly [38].

### 3.4. Sensory Aids

For individuals with sensory impairments caused by NDs, ATs provide essential support, including hearing aids, visual aids, and tactile devices. These tools enhance sensory processing, facilitate greater interaction with the environment, and promote independence. Neurorehabilitation programs frequently feature sensory technologies, with the aim of supporting sensory processing and mitigating deficits resulting from neurological damage. Recent technological advancements in electronic visual aids have led to smaller, portable, affordable, and user-friendly devices, significantly improving the lives of individuals with visual impairments. These devices include electronic magnifiers, portable reading aids, ultrasonic orientation systems, and OrCam, a discrete system that transmits visual information like text. Additionally, the evolution of smartphones and tablets has made these technologies even more accessible, replacing specialized devices at lower costs.

The importance of using these aids lies in their ability to enhance the independence and self-sufficiency of individuals with visual impairments, allowing them to perform daily activities such as reading and navigating the internet. Furthermore, these devices facilitate social inclusion by reducing isolation and providing new opportunities for access to information. Due to their affordability, users can benefit from advanced features without facing high costs. An additional crucial aspect is the social acceptance of these devices, which should be discreet to avoid stigma and encourage more active participation in society. In this context, the study by Ishigami et al., 2021 [42] examines ATs based on computer vision and image processing, focusing on devices that require coding and sensory mechanisms. These tools are essential in improving the autonomy and quality of life of people with visual disabilities. Such comparative analysis highlights the advantages and limitations of different solutions in terms of feedback, adaptability, and functionality. Proper lighting is one of the key elements: improving light quality and reducing glare and shadows in domestic environments can significantly enhance spatial orientation and safety for people with visual impairments. Automated lighting systems and colored filters applied to glasses can optimize visual perception and reduce eye strain [16]. A further useful tool is represented by prismatic glasses and telescopic lenses, which can improve visual attention and reduce the impact of visual field deficits. Digital technologies, such as magnification software and speech synthesis for computers, allow people with visual disabilities to access information and interact more effectively with the digital world [16]. Despite this, the study points out some issues, including the lack of quantitative data on the effectiveness and adoption of the devices, as well as the limited commercialization of technologies for blind people, which are often not tested in real-life conditions. The research suggests the need for strategies to bridge the gap between technological development and practical use, encouraging collaborations between companies and healthcare institutions. The integration of object and facial recognition techniques into assistive devices is promising, but their adaptability to real-world contexts remains a challenge.

In addition, the use of assistive technologies for hearing, vision, and mobility in the elderly can promote healthy aging and improve social participation. However, economic and awareness barriers hinder their spread, especially among people with lower income and education levels. Tactile communication methods, which use touch to transmit information, are fundamental as well. These systems work through vibrations perceived by sensitive areas of the body, such as the head and neck, allowing users to “read” messages through touch [43]. This method is easily learned and can be applied to practical situations, such as navigating routes or playing card games for blind individuals.

Tactile devices such as these are highly beneficial for people with visual impairments, enabling them to communicate and orient themselves without relying on vision. Moreover, these devices help them become more independent in daily life, improving their autonomy. Due to their ease of use and effectiveness in conveying information, sensory aids tools allow individuals with disabilities to have a more active participation in society, avoiding feelings of exclusion [44]. Interestingly, Sankar et al. [43] have introduced a hybrid prosthetic hand that can compliantly grasp numerous everyday objects of varying surface textures, weight, and compliance while differentiating them with 99.69% average classification accuracy. This device, as an AT, consists of a natural prosthetic hand composed of soft robotic joints and a rigid endoskeleton with three independent neuromorphic tactile sensing layers inspired by human physiology. The hybrid robotic hand with multilayered tactile sensing achieved 98.38% average classification accuracy in a texture discrimination task, surpassing soft robotic and rigid prosthetic fingers. Controlled via electromyography, this transformative prosthetic hand can aid individuals with upper-limb loss to grasp compliant objects with precise surface texture detection.

### 3.5. Brain–Computer Interfaces (BCIs)

Brain–computer interfaces (BCIs) quantify central nervous system activity and translate it into new artificial outputs that replace, restore, enhance, supplement, or improve the natural CNS outputs [45,46,47]. Based on the user’s level of intentional control, BCIs can be classified into two main categories: passive and active. This latter category allows for control of an application explicitly, via conscious control of the user’s brain activity [48]. However, active BCI systems are often limited by a low information transfer rate (ITR) and require technically demanding setups, such as the use of EEG caps. As a result, their clinical utility is currently most evident in individuals with severe motor impairments, such as those with locked-in syndrome [49]. For example, a comparative study involving individuals with severe motor impairment showed that eye-tracking systems are superior to visual P300-based BCIs in terms of both speed (ITR) and usability, while BCI systems were associated with higher cognitive workload [50].

On the other hand, passive BCIs operate by interpreting brain signals that are generated independently of any intentional user control [48]. Since they do not require active user engagement, their slower interaction speed is generally considered acceptable [51,52].

BCI could be a way to augment human capabilities by providing a new interaction link with the outside world, and is particularly relevant as an aid for disabled people. The main characteristic of BCI is the ability to distinguish different patterns of brain activity associated with a particular type of mental task [46]. This aspect is the result of a complex process that begins with brain signal acquisition, which is classified into three categories based on invasiveness: non-invasive (including EEG, fMRI, fNIRS, and MEG); semi-invasive (utilizing electrocorticography-ECoG); and invasive (employing stereo-electroencephalography–SEEG). In particular, BCIs based on EEG can use various experimental paradigms, including steady-state visual evoked potentials (SSVEP), P300 responses, mental tasks, and motor imagery [53,54,55]. SSVEP and P300 paradigms are considered passive BCIs, as they rely on external equipment to provide visual stimuli, which may lead to eye fatigue during prolonged use and diminish their practical effectiveness [56]. In contrast, BCIs based on mental tasks and motor imagery are active, requiring users to intentionally engage in complex cognitive processes, such as visualizing words being written, performing mental arithmetic, or imagining the rotation of a 3D object.

To enhance the performance of mental task-based BCIs, commonly experienced daily cognitive activities are often selected. In the motor imagery (MI) paradigm, users imagine specific movements, which elicit EEG patterns such as event-related desynchronization/synchronization (ERD/ERS) in motor-related brain regions [57]. The classification accuracy of MI-based BCIs has improved over time [58,59], leading to successful implementations in various applications. Nonetheless, this paradigm presents several limitations. Due to the limited spatial resolution of EEG, only four distinct motor imagery types, left hand, right hand, foot, and tongue, can typically be differentiated [13]. In addition, achieving reliable classification accuracy often requires extensive user training. The acquired signals undergo signal processing, which involves preprocessing (filtering, amplification, and digitization), feature extraction, and feature classification using algorithms such as support vector machine (SVM), linear discriminant analysis (LDA), artificial neural network (ANN), and deep learning (DL). Recent studies [60,61] have highlighted the effectiveness of different machine learning approaches across various BCI paradigms, including MI and P300. In particular, methods based on Riemannian geometry using spatial covariance matrices and classifiers such as SVM have demonstrated consistently strong performance. Within the DL domain, architectures like ShallowConvNet have also shown competitive results, especially when sufficient data is available to support training.

The processed EEG signals can be applied in various contexts, including the control of prosthetic limbs, wheelchairs, and VR systems [13]. However, active BCI control is still limited by low ITR and high cognitive demand, which reduces its practicality in real-world scenarios. More promising in this regard are passive BCIs, which do not rely on user intention but instead monitor brain states such as attention or cognitive workload. These systems are increasingly being explored in combination with VR to enhance adaptivity and user-centered interaction. For example, recent research has shown the feasibility of delivering P300 stimuli in VR environments using mobile platforms [62].

In patients affected by neurological disorders, BCI-robotics for motor assistance is a particular field of application of these innovative devices. They can be used for motor substitution in those cases of severe paralysis or in the case of amputation of an arm/leg. For example, invasive BCIs use intracortical recordings, which have been shown to enable neural control of a robotic arm as well as a lower-limb exoskeleton. In this sense, a case report by Hochberg and colleagues [63] was the first report on the use of invasive-BCI in a tetraplegic patient after SCI to continuously control a multi-joint robotic arm. Similarly, Collinger et al. [64] analyzed the invasive-BCI-based control of an anthropomorphic prosthetic limb with seven degrees-of-freedom (3D translation, 3D orientation, 1D grasping) in a patient with tetraplegia. After 13 weeks of training, the participant demonstrated the ability to use the prosthetic limb to perform skillful and coordinated reach and grasp movements that resulted in clinically significant gains in tests of upper-limb function. However, there has been an increased interest in designing non-invasive BCIs to control robotic arms with higher degrees-of-freedom for possible motor assistance as well as rehabilitation. Recent studies on non-invasive BCIs have reported higher-dimensional continuous motor control using novel decoding approaches as well as control strategies to tackle the low signal-to-noise ratio of non-invasive signals. In the study of Meng et al. [65], a group of healthy subjects participated in a series of longitudinal non-invasive EEG-based BCI experiments. During the experiments, the majority of the subjects exhibited improved performance over time in controlling both the virtual cursor and the robotic arm. The authors used the motor imagery paradigm and decoded the subject’s intention under the ERD/ERS framework. In paralyzed patients, Fukuma et al. [66] tested a MEG-based neuroprosthetic system to evaluate the accuracy of using cortical currents in the sensorimotor cortex of severely paralyzed patients to control a prosthetic hand. They found that patients attempted to grasp or open their paralyzed hand while the slow components of MEG signals were recorded. These results demonstrate that the slow components of MEG signals carry sufficient information to classify movement types, suggesting which type of patients could more effectively control the neuroprosthesis. As an advantage, BCIs can also provide access to internal cognitive states, such as attention, intention, or mental workload, that are not accessible through conventional control interfaces like eye-tracking or switch-based systems. While these alternative systems may offer higher ITR for users with residual motor abilities, BCIs enable a deeper integration between the user’s mental state and the assistive device, allowing for more adaptive and context-aware interaction [67,68].

In addition to mobility support, BCIs have been explored in the context of telepresence robotics. These robots, equipped with obstacle detection sensors, a camera, and a screen, allow individuals to connect with family and friends in different locations and take part in their activities [69]. Several commercial platforms already facilitate this type of interaction, including PeopleBot (Mobile Robots Inc., Amherst, MA, USA), iRobot (iRobot Corp., Bedford, MA, USA), and Robotino (Festo AG, Dietikon, Switzerland). According to Millàn et al., a key design challenge in such systems is determining who, human, machine, or both, controls the system, when control is transferred, and to what extent. There are two main approaches. (i) Systems where users manually trigger mode changes using an extra switch or button. Examples of smart wheelchairs [70] in this group include SENARIO [71], OMNI [72], MAid [73], Wheelesley [74], VAHM [75], and SmartChair [76]. However, these manual interventions can be challenging and exhausting for users who are already struggling with conventional interfaces. Adding more buttons or controls for mode selection can further complicate operation and reduce user-friendliness. (ii) Systems that rely on implicit mode changes, where the shared-control system automatically switches between modes without requiring manual user input. However, a limitation of these systems is that the mode-switching logic is hard-coded and does not adapt to individual users and their specific disabilities. In this context, Millán’s research group found a solution addressing the challenges of these two approaches. This approach asynchronously estimates the user’s mental intent and provides adaptive assistance for wheelchair navigation, significantly enhancing BCI-driven performance [46,77,78]. While asynchronous, spontaneous BCIs, such as those based on motor imagery, are often considered more natural due to their independence from external stimuli, they typically require extensive training and offer lower ITR compared to evoked BCIs [79]. In contrast, evoked paradigms such as P300-based ERP and SSVEP provide faster and more reliable control, with ERP currently representing one of the most effective solutions for active command execution. However, SSVEP may lead to fatigue or trigger epileptic seizures [53,80]. Recent developments in code-modulated VEP (cVEP) further improve performance and are increasingly gaining attention in the field of assistive mobility systems.

These systems rely on the P300 potential, an electrical brain response triggered by an anticipated rare stimulus. To generate the P300, the system randomly flashes potential target destinations multiple times, and the option that elicits the strongest P300 response is selected. The wheelchair then autonomously navigates to the chosen destination and stops, allowing the user to select a new location process that typically takes about 10 s. In addition to these paradigm adjustments, modern P300-based BCIs no longer rely on averaging multiple trials to detect the presence of a P300 response based on a predefined threshold. Instead, BCIs classify individual brain responses as either target or non-target, leveraging machine learning techniques for single-trial detection [81].

To sum up, the use of BCIs in the AT field is increasing. BCI technology offers significant advantages for individuals with neurological disorders by enabling them to control assistive devices such as wheelchairs, robotic arms, and restoring independence. These systems can enhance autonomy by allowing users to interact with their environment without relying on caregivers. However, challenges remain, including the reliability of brain signal detection, which is often affected by noise and variability, leading to errors in device operation. Moreover, BCIs require extensive training, and prolonged use can cause mental fatigue, limiting their practicality for everyday tasks. Despite these limitations, ongoing advancements in signal processing and adaptive algorithms continue to improve the usability and effectiveness of BCI-controlled assistive devices. Among these, transfer learning has been proposed as a technique to reduce the BCI calibration time [82,83], and recent developments in self-calibrating BCIs have demonstrated the feasibility of achieving usable control from the very first session without prior calibration, by leveraging error-related potentials and task constraints [84].

### 3.6. Home and Work Aids

One of the most important applications of ATs is related to home and work environmental modifications, which have revolutionized the support provided to people with motor and sensory disabilities, enabling them to maintain a high degree of independence. ATs applied to domestic and work environments offer innovative solutions to improve safety, accessibility, and the daily well-being of users with NDs. Specifically, smart homes and home automation are emerging as key tools in automating daily tasks, managing symptoms of neurodegenerative diseases like PD, and supporting people with visual neurological impairments, like MS. In the study conducted by Simonet and Noyce [85], it is highlighted how home automation and smart homes are revolutionizing the handling of PD, offering tools that go beyond traditional wearable devices. The integration of fixed sensors in domestic environments enables monitoring of clinical parameters and automated responses to emergency situations, such as falls or sudden motor crises. A crucial aspect of these technologies is their ability to provide a realistic picture of the disease in the daily context, going beyond the limitations of episodic hospital assessments [85]. One of the most promising examples can be found in sleep and movement monitoring systems, which use pressure sensors in beds to detect behaviors related to sleep disorders, such as REM sleep behavior disorder (RBD). Additionally, voice commands for activating household devices and automatic alarm systems reduce the burden on caregivers and enhance the patient’s safety [85]. People with neuro-visual deficits, meaning visual impairments resulting from acquired brain injuries or MS, can benefit from a wide range of assistive technologies and environmental modifications. Despite the numerous benefits, assistive technologies for the home and work environment still present some challenges. Simonet and Noyce [85] stress the importance of privacy protection and the management of personal data, as home monitoring systems collect sensitive information that must be handled with the highest level of security. Concurrently, the complexity and heterogeneity of the collected data pose challenges in terms of interpretation and clinical validation.

Furthermore, Copolillo and Ivanoff [16] highlight how the lack of training and familiarity with these technologies may limit their adoption among users with neurovisual deficits. Therefore, it is essential to implement training programs for users and caregivers, as well as to develop more intuitive and accessible devices [16,86]. ATs for the domestic and working environment are rapidly evolving, offering increasingly sophisticated solutions to support the autonomy of people with neurodegenerative diseases and visual impairments. The integration of home automation with traditional assistive devices has the potential to radically transform the quality of life for these users, reducing reliance on caregivers and improving the monitoring of clinical conditions [87]. However, to maximize their impact, it is pivotal to address challenges related to privacy, personalization, and user training.

Moreover, Lektip et al. [86] evaluated the effectiveness of home modifications in preventing falls among the elderly through a systematic review and meta-analysis of RCTs. Falls are a major cause of injury and death, with high healthcare costs. The study, including participants aged ≥ 60, analyzes environmental interventions such as obstacle removal and safety installations. Results show a significant reduction in falls in most studies, though adherence issues affected outcomes in some cases. The effectiveness was higher with follow-up and personalized assessments. Challenges include intervention variability, adherence difficulties, and the need for standardized guidelines. The authors recommend structured protocols, follow-up, and caregiver training to improve outcomes, emphasizing the importance of home modifications in public health strategies and hospital discharge planning. Home fall detection devices include activity recognition devices that aim to identify behaviors in which the user may be at risk of falling, or to detect falls in real-time and alert emergency personnel. It includes wearable, non-wearable, and hybrid systems with machine learning algorithms and a minimally invasive approach that becomes more accurate [56].

## 4. Discussion

In this narrative review, we sought to explore the role of ATs and related technological advancements in the field of neurological disorders. After the onset of a neurological disorder, multiple functions are often compromised, requiring not only rehabilitation but also assistive support to promote functional independence. Unlike technologies used in rehabilitation, which are primarily aimed at functional recovery during clinical treatment, ATs are specifically designed to support cognitive, communicative, and sensory functions in everyday life. It is of considerable importance that a technological device, when built to support rehabilitation, particularly in patients with severe deficits [57], is different from the device that is used to replace a function and vicariate it in the best possible way, increasing the patient’s functionality and, therefore, their quality of life. When a technological device supports rehabilitation, it is often used in the clinic, with the presence of health professionals, and has characteristics that aim at the patient’s recovery and therefore support rehabilitation [58,59]. While assistive technology devices improve function “per se”, they are often used in community life and are hopefully used without help. This difference involves technological contents, constructive principles, control methods of the device itself, and unfortunately, very often they are confused, or the same device is tried to adapt for both uses. A concrete example is the use of device feedback: if it is used for rehabilitation, feedback has a fundamental importance in the process of motor re-learning dependent plasticity, often multisensory and attentional (error based or not) and engagement; while in the case of an assistive device, feedback is important for the mere functioning of the technological device [60,61]. Their primary purpose is to promote autonomy and participation, particularly in home and work environments, across all stages of care. This raises an important question: when should neurological patients be introduced to the ATs? The answer depends on the etiology and progression of the neurological disorder. For instance, post-stroke patients might show better adherence to ATs during the chronic phase of the disease, when neuroplasticity tends to plateau. At this stage, ATs can play a meaningful clinical role by enhancing individual functional autonomy after major physical and psychological recovery milestones have been reached. On the other hand, neurodegenerative disorders such as PD, MS, and ALS tend to manifest progressive disability. In this sense, patients could benefit from tailored ATs designed according to their specific level of disability. For example, individuals with MS often present heterogeneous symptoms due to demyelinating plaques and different disease subtypes, which require personalized clinical attention. This applies not only to the rehabilitation phase but also to the support of ADLs at home and work environments, both after hospital discharge and in an outpatient care setting.

### 4.1. Multidimensionality of ATs

**Figure 1:**
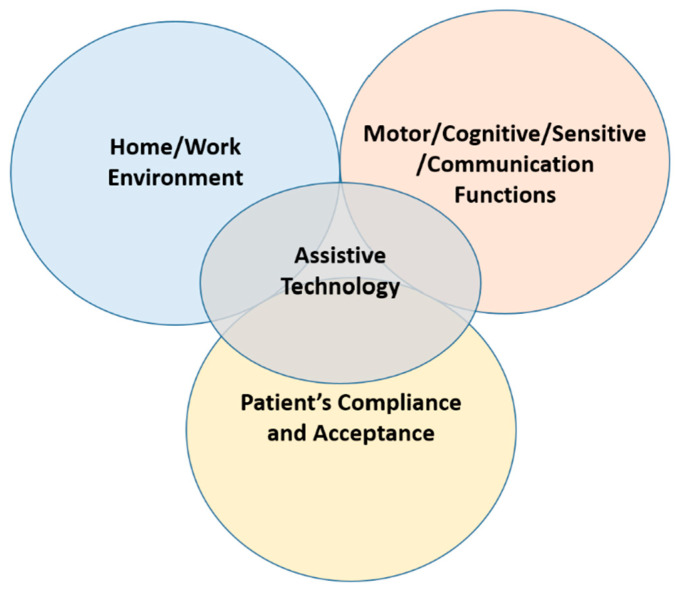
Multidimensionality of ATs.

In this narrative review, we categorized ATs based on their primary functions, such as mobility, communication, sensory, and cognitive support. However, some ATs serve multiple purposes. For example, a hybrid robotic prosthetic hand, which can be considered a BCI, may be used both for grasping (mobility) and for perceiving touch (sensory function). In this context, Luo et al. (2021) investigated the effectiveness of a bio-realistic controller combined with neuromuscular reflex control in a cable-driven prosthetic hand designed for individuals with forearm amputations [62]. Using a neuromorphic chip, the researchers successfully restored proprioceptive feedback during specific manual grasping tasks, with incremental difficulty. Their findings show that reintroducing neuromuscular reflexes through bio-realistic control significantly enhances the performance of prosthetic hands, especially in tasks requiring adaptable and compliant force modulation. Interestingly, the study also found that patients with no prior experience with myoelectric prosthetics performed even better in specific manual grasp tasks than those with previous experience. This suggests that the device is highly intuitive and requires a lower cognitive demand for the user. Despite promising results, these innovative devices are currently conceived as prototypes and are not yet suitable for use in home or clinical settings, as they are intended solely for research purposes. This represents a partial limitation, as the rudimentary design could influence patients’ perception of the devices, potentially leading to their rejection or reluctance to use them. From this perspective, ATs must be designed with a multidimensional approach, allowing the device to serve multiple functions and provide various channels of stimulation for the user. Ideally, such devices should be suitable for both home and work environments. As a result, they should promote high levels of user acceptance and satisfaction, while also encouraging patient compliance with the system (Figure 1).

### 4.2. Potential Benefits of Using ATs

Another aspect that has been only marginally explored in the literature is whether ATs can induce neuroplastic effects. According to Gandolla et al., the repetitive use of mobility-related ATs in daily life may lead to functional improvements. It is important to distinguish, however, that ATs are primarily designed to support independence in everyday activities, while neurorehabilitation tools are specifically developed to promote functional recovery [20]. In a translational sense, it should not be neglected if ATs are able to promote some enhancements in functional abilities. In this context, future research should investigate whether the repetitive use of ATs can indeed stimulate neuroplastic changes. For instance, BCIs require intensive training before they can be effectively used in everyday settings. This training often involves repetitive tasks that demand focused attention from the user, conditions that may reinforce neural connections and potentially promote neuroplasticity. Despite the promising clinical potential of ATs, they may be perceived as having a negative impact on a person’s life, particularly when the system is overly complex or not well adapted to the needs of the patient and their caregivers. Modern ATs often require substantial training for both users and caregivers. Many individuals with neurological impairments encounter difficulties in learning how to use these systems effectively, which can significantly limit their benefits. This is why these tools should be personalized according to patients’ physiognomy (e.g., mobility and BCI) and global functioning (e.g., communication, cognitive, and sensory).

### 4.3. Challenges and Potential Solutions of Implementation of ATs in Patients with Neurological Disorders

The introduction of innovative ATs into clinical practice represents a promising frontier for supporting mobility and functional independence in individuals with neurological disorders. However, the real-world implementation of these devices outside controlled research settings presents several challenges. Firstly, the usability of the systems: devices like exoskeletons should minimize complex or mechanical donning procedures, and user interfaces must be intuitive and user-friendly to avoid patient frustration and poor adherence. In this sense, the use of soft exoskeletons could represent a potential solution for their low weight, reduced dimensions, safety, and comfort, as demonstrated by Basla et al., (2022) [88]. However, there could be further challenges in its actual adoption in daily life. Major barriers included the complexity of the donning process, dependence on a caregiver for setup, and difficulties in integrating the device into an autonomous daily routine. Furthermore, a lack of intrinsic motivation and the absence of real-time feedback on activity levels further hinder consistent use. In neurological contexts, characterized by chronic conditions and heterogeneous clinical profiles, these issues become even more pronounced, requiring flexible and highly personalized solutions [88]. Another aspect that should be considered is the socio-demographic profile of potential users. Data analysis from the Canadian Longitudinal Study on Aging (CLSA) [42] shows that the adoption of ATs is more frequent among older individuals, widows, those with lower education, lower income, and poorer health conditions. Moreover, their usage varies by gender and social participation, stressing the importance of improving accessibility and information on these technologies. In this regard, it could be helpful to involve, for example, older patients in the development of specific technologies. According to Fischer et al. [89], user involvement is often recommended as a strategy to increase the relevance and usability of technological products; however, in practice, it tends to occur at low levels of participation and is often shaped by stereotypical assumptions about aging. In addition, the authors suggest that user involvement does not automatically lead to better acceptance or adoption of the technologies. In fact, the outcomes in this regard are highly variable. While some studies reported increased acceptance thanks to user-centered adaptations, others showed little to no added value in the eyes of the users, or highlighted mismatches between user expectations and the final product. In this context, Shore et al. [90] highlighted that one major barrier is the lack of user-centered design in current prototypes, which often fail to address the specific needs, preferences, and anxieties of older adults. In particular, the application of technology acceptance models (TAMs), especially those tailored to older adults, such as the Almere model and the Senior Technology Acceptance Model (STAM), offers valuable insight into the psychological, social, and functional dimensions influencing user attitudes and behaviors. These models suggest the importance of perceived usefulness, ease of use, adaptability, and social influence in promoting long-term adoption. In fact, according to user experience, the success of implementation does not depend solely on the technical performance of the device, but also on its perceived accessibility, adaptability, and relevance to everyday life.

### 4.4. Future Directions of ATs

ATs can also be used for home and work environmental control to help individuals in managing their surroundings more effectively, thereby promoting greater independence and improving their quality of life. To enhance the performance and user-friendliness of these systems, integrating artificial intelligence (AI) and machine learning (ML) into their design and development can be beneficial. While artificial intelligence (AI) and machine learning (ML) have already been integrated into some ATs, such as brain–computer interfaces, their broader application across diverse AT domains holds further potential for improving personalization, adaptability, and overall user experience. ML is a branch of AI that focuses on developing algorithms and statistical models that enable computers to learn from and make predictions or decisions based on data, without being explicitly programmed for each specific task [91,92]. In this sense, tools like remote controls, smartphone apps, or voice commands can be used to control aspects of the home/work environment, such as lighting, appliances, or motorized features. When integrated with ML, these systems can adapt to users’ routines and preferences, improving usability and personalization. For example, smart home systems can learn to optimize energy usage by turning off lights and devices when not in use or detecting faults early to prevent system failures. Furthermore, the combination of sensor-based technologies and ML has led to the development of adaptive switches. These allow users with limited mobility to control devices through alternative inputs such as touch, breath, or eye gaze, offering greater autonomy in daily activities. In addition, by analyzing data from sensors and other sources, ML can identify patterns in an individual’s movements and activities, allowing assistive devices to dynamically adjust their settings to better support the user. This is particularly relevant for intelligent prosthetics and exoskeletons, which can adapt to the user’s movements and provide real-time feedback and guidance, facilitating skill acquisition and improving mobility. Another important application of ML in ATs includes speech recognition, which enables individuals with limited ability to type or write to communicate more easily [93]. The potential of deep learning techniques to support intelligent augmentative and alternative communication (AAC) systems is explored in the review of Elsahar et al. [94]. Recognizing the limitations of conventional automatic speech recognition (ASR) systems, particularly their poor performance with non-standard speech, the authors present a personalized, speaker-dependent ASR system designed specifically for native Italian speakers with dysarthria. This system is embedded within a mobile application, which facilitates speech data collection and therapy exercises in tele-rehabilitation contexts. They developed a convolutional neural network (CNN) architecture that was used to train the speech model, which was tested in both “personal” (user-specific) and “global” (cross-user) configurations. The results show that models trained on individual user data generally outperform global models, with recognition accuracy exceeding 98% when trained with 30 repetitions per keyword. Importantly, high accuracy was achieved even among users with severe speech impairments, highlighting the robustness of the personalized approach.

Recent applications of large language models (LLMs) have opened new possibilities in AT. In particular, LLMs are a class of AI models built using advanced DL techniques, based on transformer architectures [95,96]. LLMs, such as GPT-3 by OpenAI, BERT by Google [97], Llama by Meta [98], and others, are designed to understand and generate human language. LLMs, trained on extensive datasets of human language, have developed an understanding of grammar, context, and subtle meaning, enabling them to produce text that closely resembles human communication. In this context, LLMs have recently evolved into multimodal models, which integrate multiple input types (e.g., text and images) to enhance their comprehension and output. These models aim to better align diverse data inputs with human intent, improving both understanding and text generation [96]. For example, Ray-Ban Meta smart glasses and Envision smart glasses offer promising solutions for individuals with visual impairments by converting visual input into speech [99]. The integration of GPT-4 allows users to interact more naturally with these devices, asking questions, summarizing text, or filtering information (e.g., identifying vegan options on a menu). However, the high costs of these technologies have limited their adoption.

Future research should aim to explore a multidimensional integration of assistive devices, considering not only their functional performance but also how they are perceived and experienced by users. A comprehensive evaluation of usability should encompass aspects such as user satisfaction, perceived usefulness, and the aesthetic and ergonomic appeal of the device, particularly because these technologies are intended to be used regularly in everyday life. The design of the device plays a crucial role in fostering user acceptance; if patients feel comfortable and positively connected to the technology, they are more likely to incorporate it into their routines. A greater effort must be made to evaluate the acceptance of AT. Moreover, the integration of AI and ML should be more deeply considered, especially to enhance the personalization of AT, to adapt to individual needs, preferences, and patterns of use. Finally, a systematic approach based on a multidisciplinary health technology assessment (HTA) process is mandatory to increase the diffusion of assistive devices, which must inevitably involve patient associations, and policy makers in health and public disability. They must include efficacy concerns with adequate and specific measures [68], acceptance issues, and even costs, regulatory and ethical aspects, and other issues important for their integration into the health domain [69]. From an ethical standpoint, future advancements in AI applied to ATs and neurology must consider the use of patients’ data, especially when such data are employed to train machine learning algorithms. Neuroethics requires careful evaluation of patient consent, data privacy, and the potential biases within AI models [100,101]. As AI becomes more integrated into rehabilitation settings, it is essential to implement strategies that reduce risks, support patient welfare, and prevent algorithmic bias, ensuring its responsible and ethical use in healthcare [102].

Although this review was conducted with the aim of providing a broad and informative overview of the current state of assistive technologies in neurological disorders, some limitations should be acknowledged. First, as a narrative review, the study did not follow systematic reporting frameworks such as PRISMA, which may limit reproducibility and increase the risk of selection bias. Second, only articles published in English were included, potentially excluding relevant studies in other languages. Third, we did not perform any quantitative or statistical synthesis of the findings, as the primary objective was to qualitatively explore and describe the range of assistive technologies across functional domains, their applications, and related challenges. Future systematic reviews or meta-analyses could build on this work by applying more structured methodologies and including a broader linguistic and quantitative scope.

## 5. Conclusions

The integration of assistive technologies, such as exoskeletons and BCIs, into neurology has made significant strides, improving the lives of individuals with neurological disabilities. From mobility aids to brain–computer interfaces, these technologies offer new opportunities for independence, rehabilitation, and communication. However, challenges related to usability, acceptance, accessibility, and the dosage of use remain. For instance, while BCIs are promising in supporting individuals with severe motor impairments, their practical application is still limited by a low ITR, which affects user experience and efficiency in real-life settings. Similarly, robotic exoskeletons aim to replicate natural joint kinematics. However, their practical use may be limited by factors such as the time required for donning and doffing, the considerable weight of the device, and high costs, all of which negatively impact real-life applicability and user adherence. With continued advancements in neuroscience, AI, and robotics, the future of ATs in neurology holds potential for enhancing the quality of life for individuals with neurological conditions. Future research in the field of ATs should prioritize the development of highly personalized, AI-driven solutions that can adapt in real time to users’ needs and preferences. This may include integrating ML algorithms into home and workplace control systems to enhance autonomy and usability, improving AAC and speech recognition tools through user-specific training, especially for individuals with speech disorders, and leveraging multimodal LLMs and wearable devices (e.g., smart glasses) to enable real-time interaction and contextual understanding. These advancements should be accompanied by usability assessments and robust ethical safeguards to ensure data privacy and algorithmic transparency.

To fully realize this potential, a health technology assessment (HTA) approach with collaborative efforts among researchers, healthcare providers, technologists, and engineers will be essential. HTA represents a structured, multidisciplinary process for evaluating the clinical efficacy, safety, cost-effectiveness, and social impact of healthcare technologies. In the field of ATs for neurological disorders, such an approach could ensure that innovation is aligned with real-world needs, sustainability, and ethical considerations.

## Data Availability

No new data were created.
